# Micro- and Nanosized Carriers for Nose-to-Brain Drug Delivery in Neurodegenerative Disorders

**DOI:** 10.3390/biomedicines10071706

**Published:** 2022-07-14

**Authors:** Radka Boyuklieva, Bissera Pilicheva

**Affiliations:** 1 Department of Pharmaceutical Sciences, Faculty of Pharmacy, Medical University of Plovdiv, 4002 Plovdiv, Bulgaria; radka.boyuklieva@phd.mu-plovdiv.bg; 2 Research Institute at Medical University of Plovdiv, 4002 Plovdiv, Bulgaria

**Keywords:** microparticles, nanoparticles, neurodegenerative disorders, Alzheimer’s disease, Parkinson’s disease, nose-to-brain

## Abstract

Neurodegenerative disorders (NDs) have become a serious health problem worldwide due to the rapid increase in the number of people that are affected and the constantly aging population. Among all NDs, Alzheimer’s and Parkinson’s disease are the most common, and many efforts have been made in the development of effective and reliable therapeutic strategies. The intranasal route of drug administration offers numerous advantages, such as bypassing the blood–brain barrier and providing a direct entrance to the brain through the olfactory and trigeminal neurons. The present review summarizes the available information on recent advances in micro- and nanoscale nose-to-brain drug-delivery systems as a novel strategy for the treatment of Alzheimer’s and Parkinson’s disease. Specifically, polymer- and lipid-base micro- and nanoparticles have been studied as a feasible approach to increase the brain bioavailability of certain drugs. Furthermore, nanocomposites are discussed as a suitable formulation for administration into the nasal cavity.

## 1. Introduction

In recent decades, neurodegenerative disorders (NDs) have become a global health problem due to the rapid increase in the number of people affected and the aging of the world’s population [1]. Both the direct and indirect costs of treating neurodegenerative disorders are significantly higher than those of treating cancer [2,3]. Among all NDs, Alzheimer’s (AD) and Parkinson’s disease (PD) are the most common, usually characterized by neuroinflammation, intracellular α-synuclein (α-syn) deposition, iron accumulation, increased oxidative stress, lipid peroxidation, etc. [4]. All these processes lead to neuronal damage and disrupt mobility, coordination, strength, sensation, and cognition [5,6]. Many efforts have been made to develop effective and reliable therapeutic strategies for NDs. However, most treatments focus on slowing down disease progression but do not lead to a complete cure. Many active pharmaceutical ingredients (APIs) cannot achieve the desired therapeutic effect due to the presence of limiting factors, such as the blood–brain barrier (BBB). It is, therefore, essential to ensure the safe and effective delivery of active moieties in the brain for the beneficial treatment of NDs [7].

The intranasal route of drug administration offers numerous advantages, such as bypassing the intestine, avoiding first-pass metabolism, and reducing systemic side effects. Moreover, it circumvents the BBB, providing direct entrance to the brain through the olfactory and trigeminal nerve pathways. Direct nose-to-brain delivery has emerged as an opportunity for many APIs to reach the brain but, at the same time, accomplish this is a big challenge. Factors such as mucociliary clearance, which shortens the residence time of the drug at the administration site, may compromise nasal drug delivery due to decreased nasal absorption [8].

Micro- and nanotechnological approaches were widely used to overcome these limitations and enhance the availability of drugs in the brain tissue. Micro- and nanoparticulate carriers are composed of natural or synthetic materials that interact with biological structures at the molecular level and lead the treatment of NDs into a new direction. They may induce interaction between target sites, thus minimizing the side effects.

The purpose of this review is to summarize the available information on advances in micro- and nanoscale nose-to-brain drug-delivery systems for Alzheimer’s and Parkinson’s disease.

## 2. The Nasal Route—A Shortcut to Deliver Therapeutics Directly to the Brain

The brain is undoubtedly the most protected organ in the human body from the entry of exogenous substances such as toxins and drug molecules. This protection is provided by different cells at three interfaces: the blood–brain barrier (BBB), the blood–cerebrospinal fluid barrier (BSB), and the arachnoid barrier [9]. To reach the brain, drug molecules must meet certain criteria: they should be non-ionized and lipophilic, with a molecular weight below 400 Da and capable of forming fewer than eight hydrogen bonds [10]. Most drugs used for the treatment of neurodegenerative disorders do not comply with the listed requirements for effective brain delivery. This has led scientists to search for alternative administration routes to bypass the BBB and harness the therapeutic potential of drug molecules. Therapeutics might be administered directly to the central nervous system (CNS) by intrathecal, intraparenchymal, and intracerebroventricular injections/infusions, but these routes are invasive and are not suitable for chronically administered drugs [11,12]. The nasal route has gained attention in recent years as non-invasive and easy-to-self-administer path that allows for rapid absorption and avoids the first-pass metabolism of therapeutics. The number of approved intranasal formulations is constantly growing, e.g., Rivamist^®^ (rivastigmine intranasal spray) (Lachesis Biosciences Ltd, Warrnambool, Australia), and can be used to treat agitation associated with Alzheimer’s disease. 

**Figure 1:**
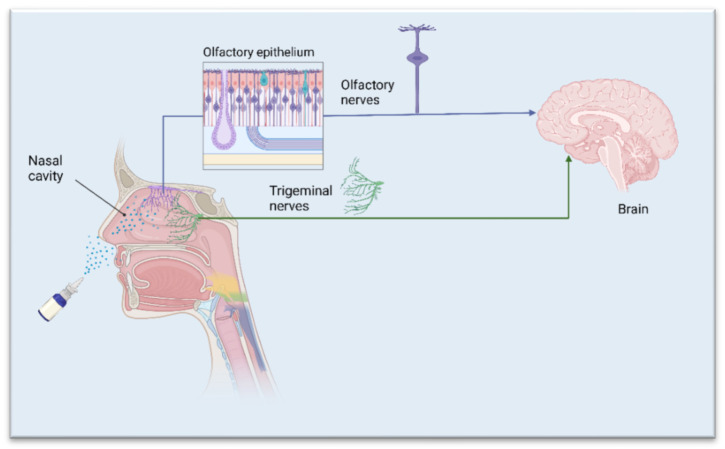
Nose-to-brain drug delivery. Schematic representation of olfactory and trigeminal neurons’ position in the nasal cavity; in purple-olfactory pathway, in green-trigeminal pathway. Created with BioRender.com (accessed on 25 June 2022).

**Figure 2:**
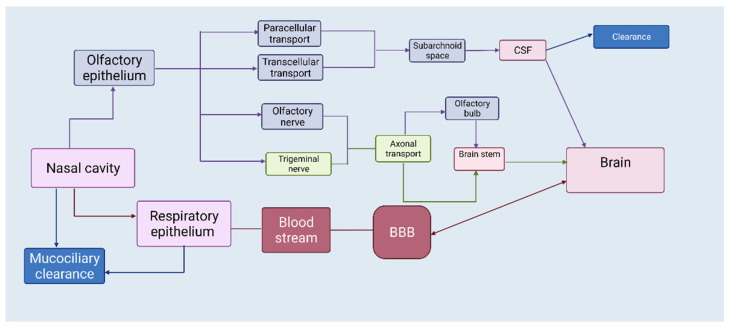
Nose-to-brain drug-transport pathways. After nasal administration drug molecules can reach the brain via olfactory, systemic, and trigeminal pathways. Olfactory and trigeminal pathways avoid first-pass metabolism of drugs and bypass BBB to deliver molecules directly to the brain via transcellular and paracellular transport. Created with BioRender.com (accessed on 25 June 2022).

Nasal formulations have the potential for self-medication and good patient compliance. The human nasal cavity extends from the nostrils to the nasopharynx (12–14 cm in length) and contains four different types of epithelia and underneath mucosa: squamous, respiratory, transitional, and olfactory [13,14]. Nasally administered drugs are deposited in the respiratory or olfactory epithelium [15,16]. From the respiratory epithelium, drugs can be absorbed in the systemic circulation, and then can reach the CNS if they can cross the BBB. Regarding nose-to-brain delivery, olfactory mucosa, and the trigeminal nerve, which innervates the olfactory and respiratory mucosa, are of particular interest (Figure 1). The nasal route provides two pathways—intracellular and extracellular—that are responsible for the drug being transported directly to the brain [14]. The intracellular pathway involves endocytosis by sensory olfactory cells, axonal transport to their synaptic clefts, and exocytosis into the olfactory bulb, where neurons projecting to brain regions repeat the process [17]. The extracellular pathway directly transports drug molecules into the cerebrospinal fluid (CSF) through the paracellular space of the nasal epithelium and then through the perineural space to the subarachnoid space of the brain [17]. Both mechanisms contribute to the transportation of drug molecules, but the intracellular pathway is quite slow and cannot demonstrate the delivery of intranasal markers to all regions of the brain far beyond the projections of the olfactory bulb. Although reinforced by limited kinetic evidence, the extracellular pathway appears to be the main component of drug transport and should be the primary target [17]. It is reasonable that a combination of these pathways may occur (Figure 2), depending on the physicochemical properties of the drugs, characteristics of the formulation, and the type of drug-delivery device. The target region for nose-to-brain delivery is the olfactory epithelium in the upper nasal cavity. Due to the rich vascularization, the nasal mucosa generally serve as an effective absorption surface for therapeutic agents. However, the olfactory region, due to its proximity to the CSF, presents a direct connection to the CNS (nose-to-brain) [18].

## 3. APIs Suited for Nose-to-Brain Drug Delivery

Dosage forms with targeted nose-to-brain delivery include mainly drugs that do not reach therapeutic concentrations in the brain tissue, otherwise administered, e.g., orally, drugs with a pronounced first-pass effect, and drugs with many peripheral side effects [19]. The main properties that affect the rate and extent to which drug molecules will be transported from the nasal cavity to the brain tissue are the molecular weight, lipophilicity, and the degree of dissociation [14].

Dopamine itself, for example, cannot be used for the treatment of Parkinson’s disease because it is incapable of crossing the BBB. In an animal study, dopamine levels were investigated in blood and cerebrospinal fluid to find out whether the drug was transferred along the olfactory pathway to the CNS following nasal administration. The drug was given intravenously or nasally. Higher dopamine levels in the brain were registered 30 min after nasal administration compared to those after intravenous administration. These results indicate that unchanged dopamine is transferred into the olfactory bulb via the olfactory pathway in rats [20]. Studies in humans have demonstrated that peptides such as melanocortin, vasopressin, and insulin, which have been shown to affect brain functions, including learning, memory, and cognition, accumulate in the brain tissue after intranasal administration [21]. Intranasal insulin was found to improve cognitive functions in patients with Alzheimer’s disease with no increase in peripheral blood levels [22]. 

## 4. Desired Features of Micro- and Nanoparticles for Successful Nose-to-Brain Delivery

Various dosage forms (solutions [21,22], suspensions [23], microemulsions [24,25], gels [26]) have been prepared for nose-to-brain delivery. Conventional forms usually do not provide a controlled release of drug molecules and are not capable of targeted delivery [27]. There is usually a rapid release and absorption of the active molecules soon after administration, and a sharp increase in plasma concentration, which can lead to toxic effects. After a relatively short period of time, this concentration falls below therapeutic levels, and this may lead to more frequent use of the dosage form [27]. Particulate formulations can offer advantages over conventional forms, such as greater stability, convenience [28] and a long residence time in the nasal cavity [29]. Another important aspect to consider when looking at nose-to-brain drug delivery is to ensure that the formulation is deposited in the olfactory region, which can be achieved with the help of appropriate devices for both liquid and solid systems [30]. Furthermore, the nasal dosage form should be designed to provide an extended residence time and maintain a high local concentration for drug diffusion [31]. Particle size is another important feature in the development of an optimized delivery system for nose-to-brain administration. Nanoparticles, for example, permeate phospholipid membranes more easily than microparticles due to their smaller size, since the tight junctions of the nasal epithelium are smaller than 15 nm. Larger particles cannot permeate the epithelium; they release the drug in the mucosal tissue, where it is usually absorbed by passive diffusion. The surface charge of the carrier plays a crucial role in prolonging the contact time between the carrier and the mucosa. Microparticles with a positive charge may adhere to the mucosa due to the net-negative charge of the mucin.

## 5. Microparticles for Neurodegenerative Disorders

Microparticles are drug-delivery systems, in the 1–1000 µm size range. They have both therapeutic and technological advantages based on their structural and functional abilities, such as modified and targeted drug delivery and release, protection of the encapsulated active agent against degradation, protection of the body from systemic side effects, dose titration and less dose dumping, more homogeneous distribution, and more predictable pharmacokinetics with reduced variables [32,33,34]. Microparticles can be considered as homogeneous or heterogeneous systems depending on the formulation and preparation process [35]. They can be incorporated in different dosage forms—liquids (solutions, emulsions, suspensions), semisolids (gels, creams, pastes), and solids (powders, granules, tablets) [33].

The deposition of particles in the human nasal cavity depends on the geometry of the nasal cavity on the one hand, and on the particles’ properties, such as size, shape, and density, on the other. Evidence in the literature suggests that particles larger than 20 µm show a preferential deposition in the anterior part of the nasal cavity on inhalation due to high inertial impaction [36], while particles smaller than 5 µm follow the airways and exit the nasal cavity [36]. Research data suggest that particles of around 10 µm in size may show a preferential deposition in the olfactory region when intranasally administered at normal inhalation rates [37]. This suggests that tailoring the carrier drug particle size (into micron-sized particles) can be a potential strategy to enhance the preferential deposition of drug particles in the olfactory region of the nasal cavity (Table 1). Since the mucoadhesive capacity is crucial for the increased residence time of drug-loaded particles in the nasal cavity, a common approach to prolonged deposition on the olfactory epithelium has been to use mucoadhesive polymers for the formulation of drug carriers [38].

### 5.1. Polymeric Microparticles

In a study performed by Gavini et al. [39], cyclodextrin (CD)-loaded mucoadhesive microspheres, used as a nasal delivery system for brain targeting, were produced by the spray-drying technique. Chitosan and alginate were used as structure-forming polymers and the in vitro capacity of cyclodextrins to interfere with the b-amyloid fibril formation was examined. In this study, none of the tested CDs (β-cyclodextrin and hydroxypropyl-β-cyclodextrin) showed direct cellular toxicity; furthermore, they both proved to have a cell protective capacity from the b-peptide. It was found that the most important factors influencing the properties of microparticles are the type of polymer, CD, and the ratio between the two. Alginate-based microspheres have a surface with multiple invaginations that provide a larger specific surface area, as well as better aerodynamic properties, than spherical particles. In addition, an interaction may occur between the alginate and calcium ions found in the extracellular fluid, which, in turn, may contribute to better adhesion. The authors conclude that the microparticulate formulation that they developed is a promising nasal drug-delivery system for targeting the brain.

Some neurodegenerative disorders are characterized by iron dysregulation [40,41]. In the CNS, iron is present in neurons, astroglia, microglia cells, and olygodenrosytes. Iron is a redox-active metal and is involved in many cellular processes, including mitochondrial respiration, DNA synthesis, myelin synthesis, neurotransmitter synthesis, and cellular metabolism [42]. Iron accumulation is a common feature of many NDs, such as Alzheimer’s and Parkinson’s disease [43]. It contributes to the progression of neuropathology via the generation of reactive oxygen species [40]. Ferroptosis, a recently discovered form of cell death, distinct from apoptosis, includes iron dysregulation, lipid peroxidation, and inflammation as major hallmarks [44,45]. Targeting ferroptosis is an attractive approach to influence NDs. Iron chelation is one of the common therapeutic strategies for Alzheimer’s and Parkinson’s disease. The chelation of excess iron should have moderate affinity to avoid the depletion of transferrin and iron-associated proteins and to prevent side effects due to iron removal in areas lacking iron overload [46,47]. Most iron chelators have low oral bioavailability and a short half-life [48]. Rassu et al. developed and characterized microparticles for the targeted nose-to-brain delivery of deferoxamine mesylate (DFO), a neuroprotector that is unable to cross the blood–brain barrier and achieve high availability in brain tissue. Spherical chitosan chloride and methyl-β-cyclodextrin microparticles loaded with DFO were obtained by spray-drying at high production yields, exceeding 50%. The drug content was close to theoretical values and the encapsulation efficiency ranged from 93 to 98%. In their research, the authors compared the properties of freeze-dried and spray-dried powders regarding their suitability for nasal administration. The results revealed that spray-drying is the preferred technique for the preparation of nasal formulations, since it allows for the preparation of smaller particles with a narrow size distribution. Moreover, microparticles based on methyl-β-cyclodextrins (MCD) appear to have great efficacy in inducing drug absorption across the neuronal component of the olfactory mucosa. Considering the poor systemic bioavailability obtained by the nasal administration of the microparticles, the MCD system appears promising for a noninvasive clinical application of DFO as a neuroactive drug in various brain diseases [49].

Another piece of research, carried out by Hussein et al., demonstrates the preparation of ropinirole hydrochloride-loaded alginate microparticles using the spray-drying technique [50]. Ropinirole acts as a dopamine receptor agonist and is used for the treatment of motor disorders in patients with moderate-to-advanced Parkinson’s disease [51]. However, ropinirole suffers from relativity low oral bioavailability due to its extensive hepatic first-pass metabolism. In addition, the oral administration of ropinirole causes gastrointestinal disturbances [51]. The incorporation of the drug into alginate microspheres resulted in improved stability and non-toxicity to an excised nasal sheep mucosa, and suggests effective delivery in the cerebrospinal fluid and the brain.

In their study, Mantry et al. also designed nasal microspheres of ropinirole hydrochloride, but the method they used was emulsion solvent evaporation. Three different mucoadhesive polymers were experimented—chitosan, carbopol 974P and guar gum—individually and in combinations. In all the developed formulations, a uniform size distribution was observed. However, the particle size was found to range from 280 ± 3.15 µm to 535 ± 2.28 µm and was considered inappropriate regarding the intended route of administration. The increase in polymer concentration leads to an increase in the size of the microspheres. Experiments were carried out with varying temperatures, stirring speeds, and polymer/emulsifier concentrations. It was concluded that temperature plays a crucial role in microparticle formation [52].

Quercetin, a flavonoid with neuroprotective function, has been studied as a potential drug for targeted brain delivery via the nasal route. Quercetin is characterized by its low oral bioavailability due to limited aqueous solubility and excessive first-pass metabolism [53]. In this case, the intranasal route is promising; however, nasal absorption is seriously influenced by the physicochemical properties of the drug and the formulation. As the volume of nasal fluids is limited, solubility is the major factor that affects the rate and the extent of nasal drug absorption. Manta et al. achieved a 17–40-fold increase in quercetin solubility via complexation with cyclodextrins (methyl-β-cyclodextrin, Me-β-CD, and hydroxypropyl-β-cyclodextrin HB-β-CD). They prepared lyophilized powders of Que-Me-β-CD and Que-HP-β-CD by freeze-drying and the results from dissolution and permeability studies were quite promising, which provided the premise for successful nose-to-brain delivery [54].

In a subsequent study, the research team blended Que-Me-CD and Que-HP-CD lyophilizates in various ratios with mannitol/lecithin microparticles (MLMPs) to form powders with improved morphological characteristics, as observed by X-ray diffractometry and scanning electron microscopy. the in vitro characterization of these powders using Franz diffusion cells revealed that the dissolution and permeation rate was from 17 to 48 times higher than that of quercetin. The ex vivo transport of powders through rabbit nasal mucosa was found more efficient compared to the pure drug. While MLMPs were prepared by spray-drying, the powder mixtures of MLMPs with Que-Me-β-CD and Que-HP-β-CD lyophilized powders were manually prepared in glass vials with a spatula in different ratios. The authors hypothesized that the presence of MLMPs would allow for easy handling of the powder, and a better positioning of the formulation in the nasal cavity. These results are very promising and provide evidence for the effective intranasal administration of the prepared quercetin formulations [55].

Rivastigmine was the focus of the work of Gao et al. [56], who produced mucoadhesive microspheres for nose-to-brain delivery in the treatment of Alzheimer’s disease. Rivastigmine is a cholinesterase inhibitor used for mild to moderate forms of Alzheimer’s disease [57]. As it struggles to cross the blood–brain barrier, its effects remain unsatisfactory. The emulsion solvent evaporation technique was used for the preparation of microspheres. The main evaluated variables were the concentration of the polymers (ethylcellulose and chitosan), the concentration of the surfactant (polyvinyl alcohol), and the stirring rate, which were critical factors for optimization of the microspheres. The surface of the microspheres was modified with lectin using a carbodiimide activation reaction. Mucoadhesive characteristics of the lectin microspheres were evaluated on goat nasal mucosa and showed good adhesion properties. In vivo behavioral and biochemical studies showed superior outcomes regarding memory retention for the lectin microspheres over plain microspheres.

In research carried out by Yarragudi et al. [58], tamarind seed polysaccharide (TSP), known as tamarind gum, was investigated as a carrier, using nasal administration to target the brain. TSP is a highly branched polysaccharide with a molecular weight of 720–880 kDa and is obtained from the endosperm of *Tamarindus indica* seeds [59]. It is composed of a β-(1,4)-D-glucan backbone with α-(1,6)-D-xylose branches that are partially substituted with β-(1,2)-D-galactose. TSP is uncharged under physiological conditions, and is therefore less likely to cause nasal toxicity. The mucoadhesion of TSP, like other polysaccharides, occurs due to interactions between the hydroxyl groups and mucin through hydrogen bonds and van der Waals attraction. Yarragudi et al. formulated TSP microparticles powder using a spray-drying technique with optimized conditions to produce 10 µm-sized particles. FITC-dextrans (5–40 kDa) were used as model drugs, which were incorporated in TSP microparticles. The size-dependent permeability of FITC-dextrans was observed ex vivo using porcine nasal mucosa. The presented data suggest that 10-µm-sized TSP microparticulate carriers have the potential to increase the residence time of drugs in the nasal cavity, to control the extent of their release, and to aid in their transportation to the brain. 

### 5.2. Lipid-Based Microparticles

A study performed by Trotta et al. describes the potential application of resveratrol in the treatment of neurodegenerative disorders via the nasal route of administration. Resveratrol is a polyphenol, stilbenoid, natural compound, that is found in different fruits, vegetables, and in red wine [60]. For central nervous system diseases, resveratrol has been reported to be effective against neurologic disorders such as Alzheimer’s [61] and Parkinson’s [62] diseases, brain ischemia [63,64] and epilepsy [65,66]. Resveratrol is characterized by its limited chemical stability, poor aqueous solubility (ca. 3 mg/100 mL) and high metabolism rate, which limit its bioavailability in vivo [67,68]. To circumvent this drawbacks, different strategies have been developed, including the use of drug-delivery systems such as liposomes, nano- and microparticles. In their work, Trotta et al. prepared resveratrol-loaded lipid microparticles (LMPs) using the melt oil/water emulsification technique, using a phase inversion procedure (aqueous phase poured into the molten lipid) to avoid loss of both resveratrol and the excipient during the particles’ formation. They also prepared LMPs coated with chitosan by adding the polymer solution to the suspension of LMPs formed during the cooling phase of the emulsion. Furthermore, a chitosan-coated LMP formulation loaded with resveratrol was prepared at a higher polysaccharide concentration. Various lipids (tristearin, glyceryl behenate, and stearic acid) in combination with phosphatidylcholine were evaluated as a surfactant. Pharmacokinetic studies indicated that the intranasal administration of chitosan-coated LMPs loaded with resveratrol produced a marked increase in the bioavailability of resveratrol in the CSF, which is expected to greatly contribute to its neuroprotective effect in the treatment of neurological disorders [69].

**Table 1:** Polymeric and lipid microparticles developed for nose-to-brain delivery in the treatment of NDs.

Active Ingredient	Polymer/Lipid	Preparation Method	Ref.
Polymeric microparticles			
β-cyclodextrin, Hydroxypropyl-β-cyclodextrin	Chitosan, Alginate	Spray-drying	[39]
Deferoxamine mesylate	Chitosan, Methyl-β-cyclodextrin	Spray-drying, Freeze-drying	[49]
Ropinirole	Alginate, Chitosan	Spray-drying	[50]
Ropinirole	Carbopol 974P, Guar gum	Solvent evaporation	[52]
Quercetin	Methyl-β-cyclodextrin, Hydroxypropyl-β-cyclodextrin	Freeze-drying	[54]
Rivastigmine	Ethylcellulose, Chitosan	Emulsion solvent evaporation	[56]
FITC-dextrans	Tamarind seed polysaccharide	Spray-drying	[58]
Lipid microparticles			
Resveratrol	Tristearin, Glyceryl behenate, Stearic acid	Melt oil/water emulsification	[69]

## 6. Nanoparticles for Neurodegenerative Disorders

The great interest in nanoparticles as drug delivery systems is due to numerous advantages such as targeted delivery of drug molecules, greater bioavailability, reduced risk of side effects, etc. [70]. Nanoparticles can incorporate both hydrophilic and hydrophobic drugs and can be used for a variety of administration routes. Inside the nasal cavity, particulates can undertake different pathways according to their size. If the size ranges between 10 and 300 nm, nanoparticles can deliver therapeutic agents through the olfactory pathway directly to the brain, if the size is less than 200 nm, the delivery will occur through clathrin-dependent endocytosis, and if it is in the range from 100 to 200 nm, the transport will occur by caveolae-mediated endocytosis [71]. Certainly, the particle size of the nanocarriers will play a crucial role in achieving brain targeting via the nasal route. However, many other factors, such as carrier type, drug properties, mucoadhesion and swelling capacity, would also be of great importance (Table 2).

### 6.1. Polymeric Nanoparticles

Polymer-based nanoparticles are colloidal systems made up of natural or synthetic polymers. One of the key features of polymeric nanoparticles, besides their higher stability in comparison to liposomes, is the variety of available polymers that can be used as carriers, and the opportunity to modify their surface to impart desired characteristics (modulated degradation rate, stimuli responsiveness, controlled drug release, etc.) [72]. Various APIs have been encapsulated in polymeric nanoparticulate systems, with the aim of improving their efficacy and stability and decreasing systemic toxicity. Bromocriptine (BRC) is an ergoline derivative and dopamine agonist that is used to treat pituitary tumors, Parkinson’s disease, hyperprolactinemia, and neuroleptic malignant syndrome. BRC has also been reported to have antioxidant effects, inhibit free radical formation, and scavenge free radicals [73]. BRC is characterized by its poor oral bioavailability and extensive first-pass metabolism; in addition, its distribution in the brain is poor, with low concentrations at the target site [74]. Md et al. prepared chitosan nanoparticles with BRC using the ionic gelation method as an approach to increase brain-targeting efficiency following nasal administration. These nanoparticles had a mean size of 161.3 ± 4.7 nm, which is considered optimal for deposition on the olfactory region in the nasal cavity. The results of this study revealed an increased brain uptake of BRC-loaded chitosan nanoparticles and improved antioxidant effects of BRC following intranasal delivery [75].

Another research team developed ropinirole hydrochloride (RH)-loaded chitosan nanoparticles using the same ionic gelation method. The size of the obtained particles was found to range from 82 to 129 nm and the mean zeta potential was +32.7 ± 1.5 mV, which demonstrated that the formulation showed satisfactory stability. Sustained drug release profiles were achieved for up to 18 h. The concentrations of RH in the brain following intranasal administration of the nanocarriers were found to be significantly higher at all timepoints compared with RH solution. The developed formulation showed the superiority of nose-to-brain RH delivery using mucoadhesive nanoparticles compared to other delivery routes [76].

The same preparation method was used by Fazil et al. to formulate rivastigmine (RHT)-loaded nanoparticles (CS-RHT NPs). Chitosan with tripolyphosphate (TPP) was used for the ionic gelation procedure and nanoparticulate systems were fabricated, with improved bioavailability and enhanced RHT uptake to the brain via the intranasal route. The mean particle size varied from 143.1 ± 9.2 to 3300 ± 7.0 nm depending on the chitosan/TPP ratio used. An in vitro drug-release study indicated a controlled and sustained release profile of CS-RHT NPs over 24 h. A marked improvement in brain uptake of CS-RHT NP was clearly observed following nasal administration [77].

A widely studied therapeutic target used in the treatment of Alzheimer’s disease is galantamine (GAL)—a tertiary alkaloid and reversible, competitive inhibitor of the acetylcholinesterase enzyme [78]. Nanaki et al. incorporated galantamine into hierarchical porous carbon (HPC) because it improved the drug loading by up to 30%. Then, GAL-HPC was encapsulated in polymeric nanoparticles, such as poly (l-lactic acid) (PLLA) and poly (lactide-coglycolide) (PLGA), to achieve control over drug release. Polymeric nanoparticles containing HPC with adsorbed galantamine were prepared by a modified double emulsification method of solid–oil–water (s/o/w). The nanoparticles were spherical in shape, with a smooth surface and no agglomeration. The size of neat nanoparticles varied between 134 and 149 nm and showed low polydispersity index (PDI) values. For all prepared nanoparticles, the zeta potential was negative and varied between −17 and −29 mV. In vivo studies showed that the intranasally administered GAL that was encapsulated in PLGA-coated carbon nanostructures was successfully delivered to the hippocampus just a few hours after a single intranasal dose [79].

Meng et al. developed Huperzine A (HupA)-loaded mucoadhesive polylactide-co-glycoside (PLGA) nanoparticles with surface modification by lactoferrin (Lf)-conjugated N-trimethylated chitosan (TMC) (HupA Lf-TMC NPs) for efficient intranasal delivery to the brain for AD treatment. Huperzine A is a naturally occurring sesquiterpene alkaloid [80]. It is a reversible inhibitor of acetylcholinesterase and improves memory in behavioral animal models [81]. NPs were prepared using the emulsion solvent evaporation method. The optimized NPs had a particle size of 153.2 ± 13.7 nm and zeta potential of +35.6 ± 5.2 mV, and sustained in vitro release over a 48-h period. The Lf-TMC NPs exhibited a longer residence time than nontargeted carriers and facilitated the distribution of HupA in the brain. The authors envisage a broad application prospect for the carriers for effective nose-to-brain delivery [82]. Chitosan nanoparticles were developed by Rassu et al. using the ionotropic gelation technique, using sodium hexametaphosphate as a safe cross-linker [83]. Genistein, a naturally occurring compound that structural belongs to a class of compounds known as isoflavones, was used as a model drug. It exerts antioxidant and neuroprotective effects [84]. Due to limited water solubility, excessive metabolism, and low bioavailability, its therapeutic potential cannot be realized. Chitosan nanoparticles were prepared and characterized for their potential for nose-to-brain delivery. The study demonstrated improved genistein penetration in vitro through the nasal mucosa compared to that with the neat drug. The absence of cytotoxicity with this carrier was confirmed in a rat pheochromocytoma-derived cell line.

### 6.2. Lipid Nanoparticles

Lipid-based nanoparticles were thoroughly investigated as drug-delivery systems and successfully entered the clinic for the delivery of small molecules. Lipid nanoparticles safely and effectively deliver drug molecules, mainly due to their easy diffusion through the nasal mucosa.

#### 6.2.1. Solid Lipid Nanoparticles (SLNs)

Solid lipid nanoparticles are a class of nanocarriers that have important advantages, such as the use of physiological lipids (e.g., solid lipid matrices composed of fats or waxes), avoiding the use of organic solvents in their preparation, the protection of sensitive drugs from the external environment (e.g., water), and the controlled release of drugs. Solid lipid nanoparticles can also be sterically stabilized, for example, by coupling with stearic acid-PEG 2000 conjugates. Sun et al. constructed a solid, lipid nanoparticle-based, in situ gel type (PAE-SLNs-ISG) drug-delivery system loaded with paeonol. Paeonol is a phenolic compound with neuroprotective functions, poor aqueous solubility and rapid metabolism in vivo. It is commonly used in Chinese medicine for NDs, such as Alzheimer’s disease. PAE-SLNs were prepared by high-temperature emulsification–low-temperature curing combined with ultrasound. PAE-SLNs had a low level of cytotoxicity; FITC-SLNs could be efficiently absorbed, and solid lipid nanoparticles had good cellular compatibility. Furthermore, the study demonstrated the effective accumulation of the in situ gel in the brain area after administration through the olfactory area, which proved to a be potentially effective strategy to realize the brain region delivery of PAE [85].

Rassu et al. proposed a novel delivery system for BACE1 siRNA, which was used for the treatment of AD, by inclusion into chitosan-coated and uncoated solid lipid nanoparticles. Due to its positive charge, chitosan ensures muco-adhesion and a prolonged residence time of the carrier in the nasal cavity. RVG-9R, a peptide derived from the rabies virus, was used to increase cell penetration, and promote transcellular transport into neuronal cells [86].

Generally, low levels of dopamine have been linked to Parkinson’s disease (PD), restless legs syndrome, and depression [87,88,89]. Dopamine (DA) depletion cannot be exogenously balanced, since it does not pass the BBB due to its hydrophilicity and ionization degree at the physiological pH [90]. Cometa et al. formulated solid lipid nanoparticles with glycol chitosan (GCS) to enhance DA brain delivery. The carriers were prepared using the melt-emulsification method. The study demonstrated that SLNs containing GCS and DA were smaller than those containing DA-loaded SLN; they had a positive surface charge and extremely high entrapment efficiency. The formulated nanocarriers showed promising characteristics for nose-to-brain delivery of dopamine from physico-chemical aspect but the authors claim that further research is needed to fully unleash their potential [91].

Another study [92] explored whether borneol can facilitate the delivery of solid lipid nanoparticles to the brain after intranasal administration. Borneol (Bo) is a bicyclic monoterpene, which is well-known in Chinese medicine to target therapeutic molecules directly to the brain [93]. The research group used Pueraria flavones (PTFs), which are known for their positive effect in the treatment of cerebrovascular disorders, such as PD and AD [94]. As PTFs have difficulty reaching the brain, Bo-modified solid lipid nanoparticles with PTF were developed using the emulsification evaporation–low temperature solidification method for successful nose-to-brain delivery. PTFs were encapsulated in three different types of SLNs: the first system contained with borneol and stearic acid (PTF-Bo-SA-SLN), the second one without SA (PTF-Bo-SLN), and the third one with only the active compound (PTF-SLN). SLNs had a mean size of 160 nm, which is considered optimal for targeted nasal administration. It was found that the PTF-Bo-SA-SLN accumulated more efficaciously in the rat brain, proving to be an excellent transport system. The same approach was studied by Song et al. [95], and remarkable targeting function to the brain was demonstrated when borneol was covalently bound to the SLN.

#### 6.2.2. Nanostructured Lipid Carriers (NLCs)

Nanostructured lipid carriers (NLCs) represent a novel group of nanosized carriers, which are composed of physiological and biocompatible lipids, surfactants, and co-surfactants [96]. NLCs hold eminent potential in pharmaceutical science due to their key attributes and extensive beneficial effects, such as an enhanced bioavailability and targeting capacity. Moreover, NLCs can more strongly immobilize drugs and prevent the particle from instability phenomena. NLCs also have advantages, including low toxicity, biodegradation, drug protection, slow release, and the avoidance of organic solvents in production [97].

As AD is considered a metabolic disorder with impaired insulin signaling in the brain, antidiabetic drugs may be an option for AD treatment. Pioglitazone (PIO) is an intensively studied antidiabetic drug for AD. It appears to cause severe peripheral adverse effects [98], so the nasal route is appropriate to avoid these. Jojo et al. encapsulated PIO into nanostructured lipid carriers, composed of tripalmitin, capmul MCM, and stearyl amine. The optimized formulation had a particle size of 211.4 ± 3.54 nm and a zeta potential of 14.9 ± 1.09 mV. Storage stability studies confirmed stability at 4 °C and 25 °C. The in vitro drug release study exhibited a sustained release of the model drug from the nano lipid carriers. The formulation exhibited improved permeability of PIO across the nasal mucosa ex vivo. Direct nose-to-brain drug transport was observed in vivo [99].

Another study demonstrated the production of lipid nanocarriers with high drug loading despite the small particle size. A combination of hot solvent diffusion and phase inversion technique was applied to obtain particles of ~20 nm in size, containing high loads of curcumin. Higher flux and permeation across porcine nasal mucosa in comparison to free curcumin was observed. Moreover, toxicity studies were performed, revealing no nasociliary damage and the intactness of the epithelial layer [100].

Nanostructured lipid carriers were developed by Rajput et al. for the nose-to-brain delivery of resveratrol in Alzheimer’s disease. The lipid carriers were prepared by the melt emulsification–probe sonication method, and further characterized in terms of particle size (132 ± 11.90 nm), zeta potential (−23 ± 3.79 mV), drug loading (9.26 ± 3.79%), and entrapment efficiency (74 ± 11.40%). The formulated lipid carriers were then incorporated into in situ gel, which showed a fivefold higher permeation across the nasal mucosa compared to resveratrol suspension. In vivo pharmacodynamic studies using a scopolamine-induced amnesia model in rats demonstrated improved memory in rats treated with in situ gel compared to orally administered resveratrol suspension [101].

**Table 2:** Polymeric and lipid nanoparticles developed for nose-to-brain delivery in the treatment of NDs.

Active Ingredient	Polymer/Lipid	Preparation Method	Ref.
Polymeric nanoparticles			
Bromocriptine	Chitosan	Ionic gelation	[75]
Ropinirole	Chitosan	Ionic gelation	[76]
Rivastigmine	Chitosan	Ionic gelation	[77]
Galantamine	Poly (lactic acid), Poly (lactide-co-glycolide)	Double emulsification of solid-oil-water (s/o/w)	[79]
Huperzine A	Poly (lactide-co-glycolide)	Emulsion solvent evaporation	[82]
Genistein	Chitosan	Ionic gelation	[83]
Lipid nanoparticles			
Paenol	Soyabean lecithin	High temperature emulsification/ low-temperature curing	[85]
BACE1 (siRNA)	Solid triglycerides	Emulsion solvent evaporation	[86]
Dopamine	Gelucire^®^ 50/13	Melt emulsification	[91]
Pueraria flavones	Borneol, stearic acid	Emulsion solvent evaporation	[92]
Pioglitazone	Tripalmitin, MCM, Stearyl amine	Microemulsification	[99]

## 7. Composites for Neurodegenerative Disorders

Polymer nanocomposites (PNCs) are a new class of reinforced materials that are formed by the dispersion of nanoscale particles throughout a polymer matrix. Nanocomposites consist of a polymer matrix embedded with nanoparticles to improve a particular property of the material [102]. Researchers span the range from the synthesis of basic structures (such as micro- and nanoparticles functionalized with molecules, simple biomolecules, or polymers) to more complex structures. The main approach initially focused on the control of shape, size, and surface charges, and then on modulating the topology of their chemical composition. At present, many biocompatible and biodegradable polymers have been experimentally and/or clinically investigated for the preparation of polymer-based composites as drug carriers [103]. By designing a composite structure, specific physicochemical and mechanical properties may be obtained. The resulting material may show a combination of its components’ best properties, as well as interesting features that single constituents often do not possess [104]. Examples of polymer micro- and nanoparticulate carriers’ applications for drug delivery in Alzheimer’s and Parkinson’s disease models are quite numerous, and they can take advantage of a relatively large number of materials that are biodegradable and suitable for particulate synthesis, including polylactide-co-glycolide (PLGA), polylactic acid (PLA), chitosan (CS), gelatin, polycaprolactone, and polyalkyl cyanoacrylates [105]. Polymeric composites might help to ameliorate the quantity and kinetic release profile of potential and existing Alzheimer’s and Parkinson’s disease drugs. The composite structure should be able, after intranasal application, to stably adhere to outer nasal olfactory epithelium to promote the release of nanoparticles loaded with active molecules. Nanoparticles should then be able to cross the epithelium and migrate to the nervous cells that comprise the olfactory nerve and project to the olfactory bulbs [13,14,15,16]. It is worth noting that the polymeric composite, after residing for a sufficient time to release its nanoparticles, should be degraded and eliminated without discomfort for the patient [106]. At present, composite structures seem to be promising drug-carriers, with numerous advantages over conventional forms, but we still need to deepen our knowledge of their properties and the peculiar features that the resulting nanocomposites are able to gain upon their carefully arranged mixture (Table 3).

Splinder et al. produced various-sized batches of PLGA nanoparticles, which were further encapsulated into a chitosan matrix to produce Nano-in-Micro particles (NiMP). The research group explored the nanoparticles’ size–time-dependent uptake mechanism into the lamina propria of porcine olfactory mucosa. Intracellular uptake was observed for the nanoparticles within 5 min of application to the epithelium. After 15 min, even larger nanoparticles associated with nuclei and neuronal fibers, suggesting effective transcellular transport within the olfactory epithelium and intracellular uptake into neuronal cells and successful delivery to the brain [107].

A recent study carried out by Dimiou et al. demonstrated the development of levodopa-loaded nanoparticles for nose-to-brain delivery [108]. Levodopa (L-DOPA) is the “gold standard” therapy for Parkinson’s disease. L-DOPA crosses the BBB to some extent, reaching the CNS, where it is converted to dopamine and provides robust relief from the motor signs and symptoms of PD [105]. However, L-DOPA has low oral bioavailability (30%) and undergoes extensive metabolism in the peripheral circulation [109]. Dimiou et al. developed a stable (for at least 5 months), 300-nm levodopa nanoparticulate formulation via encapsulation within N-palmitoyl-N-monomethyl-N, N-dimethyl-N, N, N-trimethyl-6-O-glycolchitosan (GCPQ). GCPQ-L-DOPA nanoparticles were then lyophilized overnight using sucrose as a cryoprotectant. The nanoparticles produced had a 260–280 nm size and a high positive surface charge of 40.5 mV. Steadily increasing levels of DA were achieved after nasal application of the formulation, suggesting direct nose-to-brain delivery. However, due to the pharmacokinetic characteristics of L-DOPA, indirect delivery (nasal cavity–blood circulation–brain) should not be excluded. Dimou et al. also developed GCPQ-L-DOPA nano-in-microparticles’ formulation by spray-drying. The spray-dryer settings were optimized to increase the yield of the recovered GCPQ-L-DOPA microparticles. The nasal administration of GCPQ-L-DOPA nano-in-microparticles formulation in rats leads to enhanced drug retention in the nasal cavity and effective delivery in the brain tissue, avoiding systemic side effects and allowing for the safe and effective treatment of PD.

Neuroinflammation in neurodegenerative disorders provides an opportunity for nonsteroidal and anti-inflammatory drugs to be used as preventive therapeutic agents. Nasal administration enables direct access to the CNS and limits peripheral side effects. Tiozzo et al. examined the insufflation, deposition, dissolution, transmucosal permeation, and in vivo transport to the rat brain of flurbiprofen from nasal powders [110,111]. Flurbiprofen is a chiral, non-steroidal, anti-inflammatory drug (NSAID) of the 2-arylpropionic acid class. Flurbiprofen microparticulate powders were prepared for nasal administration by spray-drying the sodium flurbiprofen solution. The liquid feed was prepared by adding NaOH 1 M to a suspension of flurbiprofen in water (2% *w*/*v*) until the drug was completely dissolved (final pH 7.40 ± 0.01). Soft pellets were prepared by spray-drying excipient microparticles of mannitol and lecithin (ratio 92:8 *w*/*w*). Soft pellets were then prepared as follows: spray-dried drug microparticles were manually and accurately mixed with spray-dried mannitol/lecithin microparticles (mass ratio 1:1). After its homogeneous drug content, the microparticle mixture was tumbled. The obtained agglomerated powder was manually sieved through a 500-µm sieve and collected on top of a 106-µm sieve. The soft pellets used for in vivo administration had a size in the range 106–500 µm. the nasal insufflation of flurbiprofen sodium powders in the form of microparticles or soft pellets constructed with excipient microparticles revealed a direct drug transport to the brain from the olfactory region. 

## 8. Future Perspectives

Particulate carriers have been reported to be effective for drug delivery via various administration routes. In the literature, many studies show that nose-to-brain delivery poses vast opportunities for targeting the brain tissue in the treatment of different CNS disorders. The delivery of drugs through the olfactory and trigeminal nerve pathways circumvents the BBB and other drawbacks associated with oral administration, such as first-pass metabolism or enzyme degradation. Thus, brain bioavailability is increased, systemic exposure is minimized, and systemic side effects are substantially reduced. Novel strategies, such as using micro- and nanosized drug carriers, have been widely explored to overcome the limitations of the nasal route of administration. These drug-delivery systems protect the drug from environmental factors, prolong the residence time, enhance drug permeability through the nasal mucosa, increase drug bioavailability, and improve therapeutic efficacy.

Regarding the treatment of neurodegenerative disorders, the number of potential biomolecules such as RNA, proteins, peptides, and monoclonal antibodies, is constantly increasing. However, their therapeutic potential is hindered by numerous limitations associated with key physicochemical and pharmacokinetic characteristics. Nose-to-brain delivery appears to be a feasible approach for the direct transport of complex biomolecules from the nasal cavity to the brain. The optimization of physicochemical properties of the drug carriers would be of key importance in this research area. Another perspective is modification of the carriers with cell-penetrating peptides or targeting ligands to improve their efficacy. The use of customized multi-targeted strategies would probably be the main focus of future research to ensure a personalized therapeutic approach. Hopefully, novel technologies that involve the use of the nose-to-brain path will allow for more novel delivery systems to enter the clinical development phase and be translated into clinical practice. 

## 9. Conclusions

This review article summarized the novel approaches based on polymer and lipid-based micro- and nanosized carriers to target the brain through the nasal cavity and their relevance in the treatment of neurodegenerative disorders, such as Alzheimer’s and Parkinson’s disease. As demonstrated by multiple studies, the excipients play a crucial role in the design of particulate formulations for nose-to-brain delivery. The promising results obtained in these studies regarding the brain bioavailability of various drugs after nasal administration give us reason to believe that new therapeutic opportunities will emerge before neurodegenerative disorders.

## Figures and Tables

**Table 3:** Advantages and disadvantages related to polymeric composites.

Advantages	Disadvantages
Biocompatible and biodegradable structures (biodegradable polymers such as PLGA, PLA, CS, gelatin, etc., are used for the preparation of composite structures). Targeted delivery (specifying the drug moiety directly into its effector site). Reduced adverse effects due to minimized systemic distribution. Mucoadhesive polymers reside for a sufficient time in the nasal cavity to release its nanoparticles. Precise control of structural characteristics. Adjustable surface modification and improved sustained release. Appropriate carrier for both lipophilic and hydrophilic drug molecules. Enhanced encapsulation efficiency. Isolation of drug molecules from the surrounding environment. Opportunity for improved gene (Si-RNA) delivery. Simultaneous use of two different drug moieties and multiple molecular targets.	Limited number of nanoparticles can be embedded into polymeric matrix primary because this may lead to a clustering effect (poor drug encapsulation). Self-aggregation may impact brain delivery. Possible unknown effects of nanomaterials on cell physiology. Potential cytotoxicity of some structures due to the low biodegradation and absorption of some materials. Bioaccumulation due to low rate of degradation; metabolism and elimination vary with the different types of materials used in synthesis.

## Data Availability

Not applicable.
